# Effect of Tamoxifen on the Management of Dopamine Agonist-Resistant Prolactinomas: A Systematic Review

**DOI:** 10.7759/cureus.35171

**Published:** 2023-02-19

**Authors:** Tuqa Bazuhair, Bakhitah Aleid, Mussa Almalki

**Affiliations:** 1 Obesity, Endocrine, and Metabolism Center, King Fahad Medical City, Riyadh, SAU

**Keywords:** prolactin secreting adenoma, resistance, cabergoline, bromocriptine, prolactin, prolactinoma, dopamine agonist resistant, tamoxifen

## Abstract

The management of dopamine agonist (DA)-resistant prolactinomas unresponsive to second and third-line treatment is challenging and requires alternative medical therapy. The presence of estrogen receptors on pituitary tumors, and the variable behavior of pituitary tumors in the presence of estrogen, prompted investigation of the role of anti-estrogen in the treatment of DA-resistant prolactinomas. The goal of this paper is to perform a systematic review of the role of tamoxifen in the treatment of DA-resistant prolactinomas.

A systematic review was conducted. Inclusion criteria were case reports, case series, and experimental studies using tamoxifen in DA-resistant prolactinomas. Exclusion criteria included review articles, DA-sensitive prolactinomas, and those that were not previously treated with DA. Data were analyzed using descriptive statistics. For continuous data, the mean was used. For dichotomous data, frequencies and percentages were used.

Data on 22 patients were extracted from the seven included studies. Twenty patients (90.9%) responded positively to the use of tamoxifen with a mean reduction in prolactin levels of 57.4%. Ten patients (45.5%) showed normalization of prolactin post-tamoxifen administration. Regression of tumor size and stability of tumor growth were reported in four out of 22 cases (18.2%). Combination therapy with DA and tamoxifen increased DA sensitivity and had a clinically significant inhibitory effect on prolactin secretion. Furthermore, tamoxifen may be considered an effective adjuvant for tumor size control. Therefore, further studies are needed to draw more clinically and statistically robust conclusions.

## Introduction and background

Prolactinomas are the most common functioning pituitary tumors subtype, accounting for approximately 50% of all pituitary tumors [1]. Unlike other pituitary tumors, it is often effectively treated with a dopamine agonist (DA) [1]. For instance, in the treatment of macroprolactinoma, the rate of tumor growth suppression and normoprolactinemia is as high as 95% [2]. However, it is known that a relatively small proportion of cases develop resistance to the initial medical therapy with DA or during the treatment course [3,4].

Prolactinomas are considered resistant to medical therapy when prolactin levels fail to normalize and tumor size is not reduced by 50% [3,5]. This presents a major challenge in the management of prolactinomas where failure to control the disease exerts unfavorable clinical outcomes. DA resistance rates are estimated at 20-30% for bromocriptine and about 10% for cabergoline [3-5].

Treatment of DA-resistant prolactinomas includes switching to cabergoline if bromocriptine or quinagolide is initially used; cabergoline dose escalation to the maximum tolerated dose; surgical resection; radiation therapy; and temozolomide (TMZ) chemotherapy in the case of aggressive and invasive tumors [3,4,6]. Although rarely encountered, failure to control the disease by a combination of the above treatment modalities has led to the introduction of various experimental treatment options. These include somatostatin analogs, estrogen modulators, and cytotoxic agents, including tyrosine kinase inhibitor (TKI) and mechanistic target of rapamycin (mTOR) inhibitors, as well as novel agents that have shown some efficacy in pre-clinical studies [1,3,6]. However, there is uncertainty about their effectiveness given limited clinical trials and conflicting results in various publications.

Understanding the underlying mechanism of DA resistance in prolactinoma may lead to the development of more targeted treatments. Notably, the role of the estrogen receptor in the development of this resistance is controversial. Some studies have shown modest reductions in prolactin levels with the use of aromatase inhibitors and estrogen receptor modulators, including tamoxifen, raloxifene, and fulvestrant [3]. However, male prolactinomas are more difficult to treat and have a worse prognosis, yet they have lower estrogen receptor expression [1,7,8].

Given this interesting finding and the various conflicting evidence supporting the use of estrogen receptor modulators for the treatment of DA-resistant prolactinomas, this paper aims to systematically review the role of tamoxifen in the treatment of DA-resistant prolactinomas.

## Review

Methodology

Study Design

A systematic review of relevant publications on the role of tamoxifen in the treatment of DA-resistant prolactinomas.

Search Strategy

**Figure 1:**
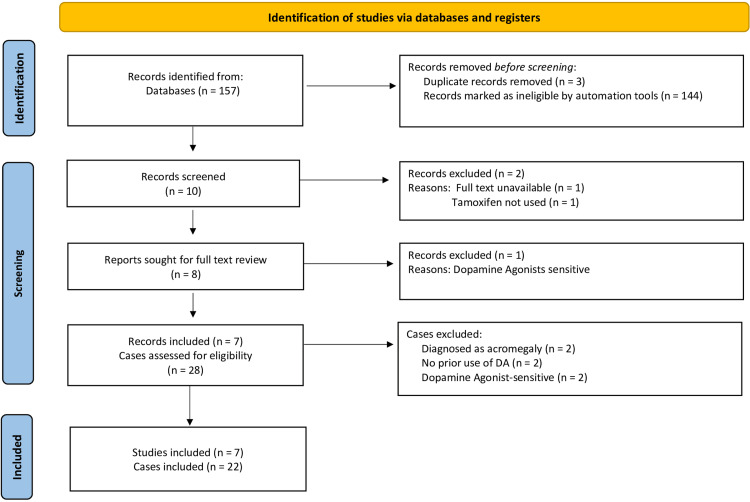
PRISMA flow diagram of search results, study selection, and inclusion process PRISMA: Preferred Reporting Items for Systematic Reviews and Meta-Analyses

A systematic search of the literature of relevant studies was conducted. Within the framework of the PRISMA (Preferred Reporting Items for Systematic Reviews and Meta-Analyses) guidelines [9], a total of seven relevant publications were identified, including case reports, case series, and experimental studies (Figure 1). It includes all relevant studies between 1980 and 2022. The following electronic databases were searched: PubMed, Google Scholar, Cochrane, and Science Direct databases. The Medical Subject Heading (MeSH) terms used in the search process included "tamoxifen", "estrogen receptor modulator", "prolactinoma", "dopamine agonist", "cabergoline", "bromocriptine", "resistant prolactinoma", "prolactin-secreting" and "pituitary adenoma". The references of identified relevant publications were searched for additional relevant articles or studies. Two reviewers (TB & BA) have independently screened the titles and abstracts of relevant publications for full-text review.

Selection Criteria

Review articles, DA-sensitive prolactinomas, and those that were not previously treated with DA were excluded. The study included published case reports, case series, and experimental studies of the use of tamoxifen in the treatment of DA-resistant prolactinomas. Publications written in languages other than English were included in the analysis following translation. Due to the varying definitions of DA resistance in the literature, all papers documenting tumor resistance according to their perspective criteria were included in the study. Studies reporting resistance only to bromocriptine were included in the analysis as well, as this occurred before the introduction of cabergoline.

Data Extraction and Quality Assessment

Extracted data included patient's age and sex; diagnosis; prolactin levels and tumor size before and after tamoxifen treatment; baseline treatment modalities before tamoxifen; tamoxifen dose; regimen of administration; duration of treatment; estrogen receptor type: ERα or ERβ, and the effect on clinical outcomes. The measurement unit of prolactin level is unified and documented as ng/ml.

For the quality assessment of included studies, we have used the Joanna Briggs Institute (JBI) critical appraisal checklist for case reports and cohort studies [10] and the proposed tool by Murad MH et al. to assess the methodological quality of case reports and case series included in systematic reviews [11].

Data Synthesis and Analysis

Given the identified heterogeneity among publications related to the treatment regimen used and the duration of treatment, the data were analyzed using descriptive statistics. For continuous data, the mean was used. For dichotomous data, frequencies and percentages were used. The results are presented as the percentage of patients achieving normoprolactinemia, the number of cases reporting a change in tumor size, the mean of the magnitude of change in prolactin levels, the percentage of patients showing improvement or resolution of symptoms, and the association of estrogen receptor subtype with treatment outcome.

Results

The systematic search identified seven studies involving the use of tamoxifen in the treatment of DA-resistant prolactinomas. Figure 1 depicts the process of study selection following the Preferred Reporting Items for a Review and Meta-analysis of Individual Participant Data (PRISMA). A full-text review of the included studies has identified a total of 22 cases that meet the inclusion criteria [2,12-16]. A total of six cases were excluded for the following reasons: The cases were not resistant to DA; DA had not been given before and two cases were diagnosed with acromegaly. Table 1 illustrates the clinical characteristics, treatment modalities, and clinical, biochemical, and structural responses of included cases.

**Table 1:** Patient’s characteristics and response to tamoxifen administration based on prolactin level and tumor NA: Data not available. ER α: Estrogen receptor alpha subtype. ER β: Estrogen receptor beta subtype. * The maximum dose of cabergoline tolerated. ** Neither the percentage nor the numerical value of the change in prolactin level was reported.

No	Author	Sex	Age (year)	Diagnosis	Baseline treatment prior to tamoxifen	Treatment regimen	Duration	Pre-tamoxifen (ng/ml)	Post-tamoxifen (ng/ml)	Change in prolactin level %	Symptoms	Tumor characteristics		Change in tumor size	ER α	ER β	Source
												Size (mm)	Invasiveness				
1	Christian, Z.K et al. [2] (2020)	F	49	Macroprolactinoma	1. Bromocriptine. 2. Cabergoline 4 mg/week. 3. Three transsphenoidal surgeries. 4. Gamma Knife radiosurgery. 5. Fractionated external beam radiation. 6. Craniotomy.	Cabergoline 4 mg/week +Tamoxifen 20 mg Three times daily	8 months	365	3	99.18	Resolved	11.5 X 15.6 X 7	Aggressive invasive tumor	No residual tumor	Absent	Present	Case report
2	Volker, W. et al. [13] (1982)	F	37	Macroprolactinoma	Bromocriptine 10 mg for 10 months + Surgery	Bromocriptine 10 mg + Tamoxifen 20 mg daily	4 weeks	70.1	23.4	66.62	Resolved	NA	Tumor grading III	NA	NA	NA	Observational study
3		F	46	Macroprolactinoma	Bromocriptine 5 mg for 5 months.	Bromocriptine 5 mg + Tamoxifen 20 mg daily	4 weeks	22.9	5.7	75.11	Resolved	NA	Tumor grade II + suprasellar extension	NA	NA	NA	Observational study
4		F	31	Macroprolactinoma	Bromocriptine 5 mg for 22 months + Surgery.	Bromocriptine 5 mg + Tamoxifen 20 mg daily	4 weeks	322	24.9	92.27	Improved	NA	Tumor grade III	NA	NA	NA	Observational study
5		F	18	Suspected microprolactinoma	Bromocriptine 5 mg for 12 months.	Bromocriptine 5 mg + Tamoxifen 20 mg daily	4 weeks	33.4	13.1	60.78	Resolved	NA	Suspected: no pituitary tumor	NA	NA	NA	Observational study
6		F	53	Suspected microprolactinoma	Bromocriptine 5 mg for 36 months.	Bromocriptine 5 mg + Tamoxifen 20 mg daily	4 weeks	56.5	12.8	77.35	Resolved	NA	Suspected: no pituitary tumor	NA	NA	NA	Observational study
7		F	31	Suspected microprolactinoma	Bromocriptine 2.5 mg for 16 months, then increased to 5 mg.	Bromocriptine 5 mg + Tamoxifen 20 mg daily	4 weeks	42.5	12.3	71.06	Improved	NA	Suspected: no pituitary tumor	NA	NA	NA	Observational study
8		F	39	Macroprolactinoma	Bromocriptine 5 mg for 12 months + Surgery.	Bromocriptine 5 mg + Tamoxifen 20 mg daily	4 weeks	58.9	43.8	25.64	No change	NA	Tumor grade II	NA	NA	NA	Observational study
9		F	29	Macroprolactinoma	Bromocriptine 5 mg for 25 months.	Bromocriptine 5 mg + Tamoxifen 20 mg daily	4 weeks	33	43.9	-33.03	No change	NA	Tumor grade III	NA	NA	NA	Observational study
10		F	27	Macroprolactinoma	Bromocriptine 5 mg for 10 months.	Bromocriptine 5 mg + Tamoxifen 20 mg daily	4 weeks	152	134.5	11.51	No change	NA	Tumor grade I	NA	NA	NA	Observational study
11		F	24	Macroprolactinoma	Bromocriptine 5 mg for 6 months.	Bromocriptine 5 mg + Tamoxifen 20 mg daily	4 weeks	22.6	44.3	-96.02	No change	NA	Tumor grade I	NA	NA	NA	Observational study
12	Koizum, K. et al. [16] (1986)	F	26	Microprolactinoma	Bromocriptine 10 mg.	Bromocriptine 10 mg + Tamoxifene 20 mg daily	1 month	61	NA	Normalized**	Resolved	NA	Non-invasive	NA	NA	NA	Case series
13		F	25	Macroprolactinoma	Bromocriptine 10 mg.	Bromocriptine 10 mg + Tamoxifene 20 mg daily	2 months	71	15.2	78.59	Resolved	NA	Non-invasive	NA	NA	NA	Case series
14	Lamberts, S. et al. [15] (1980)	F	41	GH-Prolactin co-secreting pituitary tumor	Bromocriptine 10 mg (reached a max of 30 mg).	Bromocriptine 10 mg + Tamoxifene 20 mg daily	3 months	21	11	47.62	Resolved	NA	Non-invasive	No change from the baseline	NA	NA	quasi-Experimental study
15	Gollard, R. et al. [14] (1995)	F	33	Prolactin-secreting pituitary carcinoma	1. Bromocriptine 2.5 mg/daily. 2. Transphenoidal surgeries, twice. 3. Proton-beam radiation therapy. 4. Fractionated external beam radiation. 5. Chemotherapy (cisplatinum and etoposide) 2 cycles. 6. Chemotherapy (procarbazine, lomustine, vincristine) 2 cycles.	Bromocriptine stopped Tamoxifen 10 mg twice daily	10 months	6486	NA	NA	Stable	NA	Invasive tumor with metastasis	Shrinkage of disease in the sella and temporal lobes and stability of other lesions	NA	NA	Case report
16	Lopez, J. et al. [12] (2005)	F	32	Microprolactinoma	1. Transsphenoidal surgery. 2. Lisuride. 3. Bromocriptine. 4. Quinagolide. 5.Cabergoline.	Tamoxifen 20 mg daily one month later Bromocriptine 5 mg daily added Bromocriptine increased to 7.5 mg daily	2 months	293	118	59.73	Resolved	0.9	Non-invasive, no residual post-surgery	Stable	NA	NA	Case report
17	Fedorova N. S. et al. [17] (2017)	F	26	Macroprolactinoma	Cabergoline 3.5 mg/week.	Cabergoline * + Tamoxifen (initiated at 20 mg and escalated to 40 mg after 1 month if no response is seen]	3 months	85.5	NA	32.00	Resolved	NA	NA	NA	NA	NA	Observational study Prospective single-center clinical study
18		F	33	Macroprolactinoma	1. Cabergoline 11 mg/week. 2. Transsphenoidal surgery. 3. Radiation therapy.		3 months	74.4	NA	38.00	No change	NA	NA	NA	NA	NA	
19		F	29	Macroprolactinoma	Cabergoline 2.52 mg/week.		3 months	104	NA	45.00	Resolved	NA	NA	NA	NA	NA	
20		F	23	Macroprolactinoma	1. Cabergoline 23 mg/week. 2. Transsphenoidal surgery.		3 months	158	NA	22.00	Resolved	NA	NA	NA	NA	NA	
21		F	38	Macroprolactinoma	Cabergoline 7 mg/week.		3 months	243	NA	66.00	No change	NA	NA	NA	NA	NA	
22		F	24	Macroprolactinoma	1. Cabergoline 3.5 mg/week. 2. Transsphenoidal surgery.		3 months	526	NA	64.00	No change	NA	NA	NA	NA	NA	

Quality Assessment

The overall quality of the cases is good, given that most of the cases fulfill the criteria of the quality assessment tool used. All cases reported sufficient description of the patient demographics, clinical history, pertinent investigation results, and baseline treatment. An adequate description of exposure and outcomes was provided. The length of follow-up was appropriate to assess the desired outcome. In some cases, the dose-response effect was assessed. All cases reported information on the presence or absence of side effects.

Patient's Demographics and Baseline Treatment Modalities

All patients included are females. The mean age of these patients was estimated to be 32 years, with a range of 18 to 53 years. The analyses identified 15 cases of macroprolactinomas, two cases of microprolactinoma, one prolactin-secreting pituitary carcinoma, one GH-prolactin-secreting pituitary adenoma, and three cases of suspected microprolactinoma based on a negative CT scan, although the clinical and biochemical behavior represents a probable microadenoma. These tumors were mostly invasive in nine cases (40.1%). The baseline treatment modalities include medical therapy with DA, surgical resection, and radiation therapy of both stereotactic and conventional types. In addition, pituitary cancer patients received four cycles of chemotherapy before tamoxifen. Regarding DA, 15 cases received bromocriptine at a mean dose of 5 mg. The minimum dose used is 2.5 mg per day and the maximum dose is 10 mg per day. Cabergoline was used in eight patients with a mean dose of 8 mg per week. The minimum dose of cabergoline used is 2.5 mg per week and the maximum dose is 23 mg per week. Information on the baseline medical therapy was inconsistently reported among the included studies. Surgical resection was performed in nine cases, most of them using the trans-sphenoidal approach. Radiation therapy was given to three patients as follows: one patient received both stereotactic and external beam radiation, one patient received stereotactic radiation therapy only, and one patient received proton beam radiation therapy and fractionated external beam radiation.

Administration Regimen of Tamoxifen

There were three regimens observed: (a) co-administration of tamoxifen with DA, where it was given to 14 patients in combination with bromocriptine for one to 10 months, and in combination with cabergoline in seven patients for up to eight months. (b) Tamoxifen was given as monotherapy for 10 months after discontinuing DA in one patient with prolactin-secreting cancer. (c) Pre-treatment with tamoxifen alone for one month followed by the addition of DA in one patient with microprolactinoma. The dose of tamoxifen used ranges from 20-60 mg per day. In the majority of patients (16 of 22), 20 mg/day is prescribed. In six of twenty-two patients, tamoxifen was started at 20 mg and increased to 40 mg daily if no response was observed after one month of treatment. In one patient, tamoxifen was initiated at 20 mg three times per day and then decreased to 20 mg twice daily after eight months of normal prolactin levels.

Change in Prolactin Level

Twenty patients (90.9%) responded well to tamoxifen. The levels of prolactin decreased by an average of 57.4%, with a range of 11.5% to 99%. Ten patients (45.5%) showed normalization of prolactin after tamoxifen administration. Combination with cabergoline reduced prolactin levels by 52.3% while tamoxifen in combination with bromocriptine reduced prolactin levels by 60.6%.

A sub-analysis of the effect of tamoxifen on prolactin level based on prior treatment with radiation therapy and/or surgery revealed a reduction of prolactin by 68.6% in two patients who had previously undergone surgery and radiation therapy and a reduction of prolactin by an average of 58.4% in patients who had previously undergone surgery.

Change in Tumor Size

The analysis was limited by significantly missing data from 18 patients. However, regression of tumor size and stability of tumor growth were reported in four out of 22 cases (18.2%). One case showed complete resolution of an invasive macroprolactinoma after eight months of combined cabergoline and tamoxifen therapy. The second case demonstrated the stability of a noninvasive GH-prolactin co-secreting pituitary tumor after three months of combined treatment with bromocriptine and tamoxifen. Despite a suboptimal prolactin level, a case of microprolactinoma with no residual tumor after surgery showed no tumor recurrence at follow-up. Metastatic pituitary carcinoma showed interval stability at 10 months of follow-up.

Estrogen Receptor Status

Histopathologic examination of the tumor was reported in only one case, in which prolactinoma with ERβ-positive and ERα-negative staining was identified. The patient received cabergoline and tamoxifen combination therapy and showed normalization of prolactin levels with a resolution of the residual tumor.

Discussion

DA-resistant prolactinomas represent a challenging clinical encounter, especially when they don’t respond to the second and third-line treatment modalities. As an additional measure, based on evidence from in vivo and in vitro studies and case reports, anti-estrogens have been proposed as an intervention with the potential to reduce prolactin levels and tumor size [3]. Anti-estrogens used to treat DA-resistant tumors include raloxifene, tamoxifen, and fulvestrant [3]. Current evidence suggests that the administration of raloxifene results in a small, clinically insignificant decrease in prolactin secretion [18], whereas the evidence for the use of fulvestrant has not been established in clinical trials [3]. However, the evidence regarding the effectiveness of tamoxifen is conflicting between different studies in the literature. Therefore, this paper provides a systematic review of the literature to provide evidence for the role of tamoxifen as an effective treatment modality for the management of DA-resistant prolactinomas.

The rationale for evaluating the effect of anti-estrogens on prolactinomas stems from the following findings: although pituitary tumors have been found to express estrogen receptors [8,19], some studies have shown no change in the size or behavior of pituitary tumors when exposed to high estrogen levels such as during pregnancy or exogenous steroid administration [13]. In contrast, some studies demonstrated excessive tumor growth under high endogenous estrogen during pregnancy [13]. Furthermore, although prolactinomas are known to express lower estrogen receptors in men, aggressive prolactinomas are more frequently observed in male patients than in female patients [7,13].

The results of this systematic review demonstrate that tamoxifen enhances the response to DA. In 90.9% (20/22) of DA-resistant prolactinomas treated with tamoxifen, prolactin levels were reduced by an average of 57.4% from baseline. This is consistent with the findings of in-vitro studies in which tamoxifen increased the sensitivity of scattered prolactinoma cells to dopamine and bromocriptine [20].

Furthermore, the response seen with tamoxifen monotherapy in a patient with prolactin-secreting cancer suggests that tamoxifen might have a direct effect on prolactin secretion that is independent of DA action, yet the magnitude of this effect remains to be elucidated. In comparison to the literature where experimental studies have shown an acute inhibition of prolactin release within five days of tamoxifen administration [15,19], this study highlights the ability of tamoxifen to normalize prolactin level with chronic co-administration with DA.

Although the reduction in prolactin level in combination therapy with tamoxifen and bromocriptine appeared to be greater than that with cabergoline, 60% vs. 52%, this is not conclusive evidence considering that more patients used bromocriptine and the duration of treatment was not comparable.

In vivo and in vitro studies have demonstrated a beneficial effect of tamoxifen on tumor size [1,3,21]. The results from this study showed that four of 22 patients had stability, regression of tumor size, and resolution of a residual pituitary tumor. Data were missing in 18 cases, possibly due to the short duration of tamoxifen administration to assess the effect on tumor size. Nevertheless, it is reasonable to consider tamoxifen as an effective adjuvant for tumor size control, especially when considered in the context of in-vitro studies [22,23], the missing data possibly caused by the short-term use of tamoxifen. For example, a study of tamoxifen in estrogen-induced, transplantable, prolactin-secreting rat pituitary adenomas significantly reduced tumor size when administrated shortly after tumor transplantation, whereas it inhibited the growth of the tumor when given after a long time has elapsed from transplantation [23]. The postulated mechanism for the impact of tamoxifen on tumor size includes both a direct toxic effect on the tumor cells and anti-estrogenic properties, which enhance the sensitivity of the cells to the apoptotic effect of dopamine [2,22].

This study identified three different dosing regimens. The first involved the use of 20 mg per day as a single dose or as two divided doses per day. The second regimen escalated the dose to 40 mg daily if no response to treatment after one month. The third regimen involved using 20 mg three times a day. The third approach resulted in a more significant reduction in prolactin levels and tumor size. This finding may indicate a dose-dependent effect of tamoxifen, similar to what was found in both in-vivo and in-vitro studies [22]. However, since the dose escalation approach was only employed in a few cases, more research is needed to draw a more reliable conclusion.

The long-term safety of tamoxifen in the treatment of DA-resistant prolactinomas is inconclusive considering that, in most cases, tamoxifen is only used for a short period of time. However, this study did not show any major adverse events in patients of different ages, doses, tamoxifen administration, and combination regimens. In addition, the study by Fedorova et al. evaluated the safety of tamoxifen in terms of endometrial thickness and biochemical profile, which comprised a complete blood count, liver function test, lipid profile, and renal function test, whose results revealed no significant abnormalities [17]. Of note, in one case, the dose of tamoxifen administered exceeded the Food and Drug Administration (FDA)-approved dose of 20-40 mg per day for the treatment of breast cancer [24]. This study included four patients who were reported to have had uneventful pregnancies and fetal outcomes with tamoxifen [16,17]. However, more safety data are available from studies evaluating the long-term use of tamoxifen in breast cancer.

Identifying predictors for favorable outcomes versus poor response to combination therapy may help select patients for tamoxifen-DA combination therapy. Expression or absence of estrogen alpha (ERα) or beta (ERβ) can be used as a predictor for both tumor aggressiveness and response to therapy. In contrast, age, sex, tumor type or invasiveness, prior irradiation, and surgeries were not associated with response to the combination therapy.

In Lambert et al.'s study, it was suggested that prior radiation therapy was a limiting factor in the effectiveness of tamoxifen, as they observed no change in prolactin levels in three patients who had received prior radiation therapy [19]. However, this study identified three cases of clinically significant effects of tamoxifen despite receiving prior radiation therapy within two to seven years [2,14,17]. Therefore, prior radiotherapy cannot be considered a limiting factor.

It has been postulated that the expression of estrogen receptor subtypes ERα or ERβ is a predictor of tamoxifen response [2,4,7,21]. ERα variants may play a role in tumorigenesis and the development of DA resistance while ERβ may be a modest modulator of downstream reactions in the presence of ERα and a more predominant modulator in the absence of ERα [2,7]. It has been established that there is an interaction loop between the prolactin receptor and ERα, through which the resistance to DA is augmented, resulting in a more aggressive tumor [21]. Estrogen upregulates the prolactin receptors (PRLR) on pituitary tumor cells through ERα, so the binding of prolactin to the PRLR induces phosphorylation of ERα, leading to a cascade of molecular reactions causing DA resistance and promoting pituitary tumor enlargement and invasiveness [21]. In addition, tamoxifen has pure ERβ antagonist properties compared to the partial antagonist and agonist properties for ERα [2]. Thus, it disrupts the PRL/ER crosstalk by blocking the estrogen receptors [2,21]. This mechanism may explain the positive response to tamoxifen observed in this study, in which a patient with an ERβ-positive prolactinoma exhibited the greatest reduction in prolactin level and tumor shrinkage in response to tamoxifen treatment.

Male prolactinomas are often found to be invasive and more resistant to DA [7,8]. It is proposed that the absence of ERα in male prolactinoma is associated with poorly differentiated tumors, similar to what is observed in breast cancer [7]. However, the reason why some male patients respond to tamoxifen therapy is explained by the predominant action of ERβ in the absence of ERα [7]. Notably, there were no male patients in this study.

Compared with other anti-estrogens, this study showed that the reduction in prolactin levels with tamoxifen combination therapy was greater than that achieved by raloxifene. It is more clinically significant, with a resolution or improvement of symptoms in 68.2% of patients. Comparison with fulvestrant is difficult due to the lack of studies in human-prolactinomas [4,6].

## Conclusions

This study highlights that tamoxifen enhances the inhibitory effect of DA on prolactinomas. Its effect on tumor growth inhibition and regression is direct and anti-estrogen mediated. Differences in response between cases can be attributed to differences in tamoxifen doses, administration regimen, and tumor molecular properties. The results of this review encourage the use of tamoxifen and DA in combination therapy in patients with DA-resistant prolactinomas refractory to standard treatment modalities.
